# Suppression of Perspiration

**Published:** 1873-06

**Authors:** 


### Suppression of Perspiration.

Socoloff gives an abstract of the results which follow varnishing the skin and suppression of the cutaneous secretion:

1. A few hours before the death of the animals so treated, clonic and tetanic spasms appear in various groups of muscles, while the temperature in the rectum sinks in a marked degree.
2. Enveloping the animals in wadding did not serve to raise the temperature or arrest the fatal result.
3. Respiration of oxygen proved ineffectual to resuscitate the animals.
4. In the stomach ulcers were observed, the result of deep extravasations.
5. Albumen appeared in the urine very soon after the skin was varnished.

6. In all cases a diffuse parenchymatous inflammation of the kidneys was observed—sometimes swelling of the cells, and sometimes fatty degeneration. This result was independent of the nature of the varnish used, whether turpentine varnish, or gelatin, or gum.

Lang (*Arch. d. Heilkunde,* xiii., pp. 277–287, 1872) investigates the causes of death when the skin has been varnished. In addition to other phenomena he found an hour or two after death "triple phosphate crystals" in various parts of the body, and some of the uriniferous tubules blocked with a finely granular dark mass. He thinks that the triple phosphate crystals are the result of decomposition of urea, and that the cause of death is uræmia.—*Journ. Anat. and Phys.*, November, 1872; from *Centralblatt,* No. 44, 1872. —*Am. Jour. Med.*

